# Finding the common good in divided societies: The benefits of self-interest in transitional justice interactions

**DOI:** 10.1177/13691481251356805

**Published:** 2025-07-27

**Authors:** Ivor Sokolić, Venera Çočaj

**Affiliations:** 1 University of Hertfordshire, Hatfield, UK; 2 European Institute, London School of Economics and Political Science, London, UK

**Keywords:** transitional justice, former Yugoslavia, Conversation Analysis, deliberative democracy, self-interest

## Abstract

Post-conflict transitional justice aims to nurture constructive dialogue between members of formerly opposed groups. Expressions of self-interest are often deemed antithetical to this because they can be framed as interest pertaining to a single group. We argue that expressions of self-interest can be beneficial to transitional justice dialogues. In sequences of interactions between individuals across group lines, expressions of interest are dynamic and can shift. We analyse how they can shift and how they result in a common good orientation based on a shared vision of justice. We draw on theories of self-interest in deliberative democracy and use Conversation Analysis, a micro-analytic approach to the analysis of turns in conversations, to capture this in transcripts from 12 multi-ethnic focus groups conducted in four former Yugoslav countries. The findings show that expressions of self-interest in transitional justice dialogues are more complex than previously theorised and can aid transitional justice.

One of the key aims of transitional justice is to rebuild relationships between formerly opposed groups (Mani, 2002: 15). This occurs at the micro level, between ordinary individuals in their face-to-face discussions, as much as it does at the level of elites and institutions. Constructive dialogue and interaction between members of different groups, over topics related to a difficult past, foster transitional justice (Nadler et al., 2008). There is an expectation that when transitional justice works, former antagonists will cooperate and engage harmoniously in their interactions focused on past violence (Fisher, 2001: 26). Within this context, expressions of self-interest – expressed as the interests of a specific group – are seen as counterproductive to the process of transitional justice, because they can be framed as divisive statements that further deepen divisions between groups.

Discursive expressions of interest are linked to social identity and can help explain conflicts based on group membership; discrimination; political campaigns; and, the formation of human capital (Chen and Sherry, 2009: 432). By examining expressions of self-interest, we gain a better understanding of how groups define themselves, how they adopt behaviours and how they interact with other groups (Chen and Sherry, 2009: 432). In order to create and maintain social identity, individuals can use several discursive strategies (Everett et al., 2015). First, they can attempt to deny belonging to a group they deem inferior. Second, they can construct a discourse in which their own group is presented in positive terms, especially in comparison to another. Third, they can actively attempt to change the standing of their social group. Across all three strategies, one of the most common ways to preserve social identity is to systematically evaluate one’s own group more favourably than an outgroup (Tajfel, 1982). Within these contexts, expressions of self-interest help individuals define their group, which can result in intergroup bias (Tajfel, 1982).

We argue that expressions of interest, including self-interest, are more dynamic and complex than previously theorised. The current scholarship has not captured this because it has been unable to conceptualise the dynamic nature of human interaction. Studies examine statements made by individuals in isolation from other statements, such as responses, questions, and gestures. Such an approach both theoretically and methodologically oversimplifies the richness of human interaction in the real-world setting, including expressions of interest. We address this theoretical limitation by drawing on theories of deliberative democracy that help analyse how communication can induce reflection on preferences, values and interests in a non-coercive fashion (Dryzek, 2002: 76). We show that in transitional justice, as in deliberation, self-interested statements can provide information on common interests and can help clarify conflict (Mansbridge et al., 2010). This is a way for individuals to seek a genuine common good through the negotiation of conflicting interests (Mansbridge et al., 2010: 93). Self-interest can result in, ‘transformations of preferences, and even on rare occasions transformations of underlying identities, in the direction of a common good’ (Mansbridge et al., 2010: 79). In transitional justice, this common good orientation takes the form of a vision of justice that goes beyond a single group.

We address the methodological limitation of the current scholarship, which oversimplifies the dynamic nature of human interaction, by analysing original transcripts drawn from 12 multi-ethnic focus groups composed of young people in Bosnia-Herzegovina, Croatia, Kosovo and Serbia, all focused on the topic of transitional justice. We apply Conversation Analysis, a micro-analytic method to analyse sequences of interactions (Heritage and Clayman, 2010). This allows us to empirically show how expressions of self-interest in discussions of transitional justice across group lines can help bring groups together, rather than divide them.

Expressions of self-interest are prone to change in interactions between individuals across group lines, just as ethnic lines, and in doing so they can result in orientations towards the common good. The common good orientation is a broader, more universal, vision of justice that goes beyond a single group. We propose that self-interested statements, even when made in the context of transitional justice, can promote transitional justice by providing information on common interests. When individuals express self-interest they can show how transitional justice or injustice affects them personally, as well as their group. This helps all parties involved in the interaction to orientate towards the common good. We ask, how can expressions of group interest result in common good interest? This is based on the assumption that expressions of interest are not stable, so we also examine how expressions of interest shift during inter-ethnic interactions. The expressions and shifts are significant, since they show that processes of transitional justice are more dynamic at the micro level than currently theorised.

The paper contributes to the scholarship that views post-conflict transitional justice as a process that attempts to instil changes at the everyday cultural and political levels (Gready and Robins, 2014). Transitional justice viewed this way focuses on changing discourses and interactions among and between ordinary individuals in the aftermath of conflict (Andrieu, 2010). The paper makes three contributions. First, we dispute the literature that argues that inter-ethnic dialogue restates and exacerbates ethnic tensions (David, 2020). We support our argument using a novel theoretical framework and methodological approach, which we apply to empirical data collected from inter-ethnic focus groups and show that inter-ethnic dialogues can be beneficial to the transitional justice process. Second, we show that shifts towards the common good in interactions across group lines are an observable indicator of how transitional justice works at the micro level. A focus on the common good shows that individuals are prepared to discuss a vision of justice that benefits all of society, not only their own group, which is often overlooked in the transitional justice scholarship (Dworkin, 2010: 413). The pursuit of the common good reflects a shared moral and political responsibility among individuals, which maximises the potential for peaceful coexistence (Villa-Vicencio, 2006: 393). Third, our findings can help practitioners working in conflict contexts to better manage dialogues by identifying the type of self-interest statements that aid the transitional justice process.

The paper next outlines the existing scholarship on inter-group dialogues and proposes a theoretical framework that can better take into account the dynamic nature of interaction and expressions of self-interest. It then provides an overview of a complementary methodological framework that can capture discursive shifts towards the common good. The empirical analysis that follows is based on focus group data collected from Bosnia-Herzegovina, Croatia, Kosovo and Serbia. This data is used to show how expressions of interest are not stable, instead they are dynamic and they shift. These shifts may result in a common good orientation.

## Self-interest in the aftermath of conflict

In transitional justice, statements based on group interest or self-interest can be the beginnings of antagonistic interactions, because they can frame interactions as ‘us versus them’ (Bergholz, 2016: 694). Statements oriented towards the common good, on the other hand, explicitly reject such a framing. The suppression of self-interested statements can, however, also suppress the expression of individual or group needs. Self-interested statements can provide information on the common good just as much as statements oriented towards the common good (Joshua Cohen and Rogers, 2003: 247). Individuals should be able to express these types of interest to show how transitional justice affects them personally or their group (Mansbridge et al., 2010: 73). They should also be able to hear others express their interest, to understand how transitional justice processes are affecting them. Without this exchange of information, there is a risk of a collective adopting a version of the common good that does not take everyone’s interests into account (Mansbridge et al., 2010: 73). Expressions of interest across group lines can thus result in different outcomes than the literature on transitional justice assumes, such as building a basic understanding of an outgroup, providing recognition of victimhood and advocating minority interests.

We focus on ethnic interest in our analysis because ethnic identity was the key group marker in the Yugoslav conflicts.^1^ We define ethnic interest as a statement that expresses the interests of an individual based on group belonging (Fung, 2003: 344). The articulation of self-interest can take the form of testimonies, story-telling, relating needs, advocacy and the expression of disagreement; all expressed on behalf of an ethnic group. We define the common good orientation as a vision of justice that is expressed in relation to a group greater than only an ethnic group, for example, a multi-ethnic state, a region, a generation, and humanity (Dworkin, 2010: 413). We argue that expressions of ethnic interest in discussions of transitional justice can provide information on the common good and result in orientations towards the common good. The literature on inter-group dialogues has so far ignored this because it has theoretically and methodologically (discussed later in the paper) oversimplified the nature of human interaction. The findings travel beyond merely ethnic conflicts, since they speak to broader group identity dynamics.

## Self-interest, deliberative democracy and inter-group dialogues

Much of the scholarship on inter-group dialogues assumes that they can only have positive outcomes if there is a pre-existing consensus about transitional justice among individuals or if they interact under highly specific circumstances, such as in the context of truth and reconciliation commissions (Bohm, 2004; Corry, 2012; Fisher and Keashly, 1988; Rabinowitz, 2001; Saunders, 2000). Alternatively, other scholars argue that discussing transitional justice topics across group lines only exacerbates ethnic tensions (Bashir and Goldberg, 2014; Bergholz, 2016; David, 2020; Horowitz, 2001: 7; Nwogu, 2010).

The two schools of thought share a commonality; they both assume that social interactions are static. They assume that a common good orientation can only be the outcome of consensus or already existing shared interests. They do not envisage common interest formation through a process of interest clarification, which may involve expressions of self-interest. They oversimplify human interaction and transitional justice at the micro level, and ignore the potential for social change to occur through inter-group dialogues.

Our study adds to the nascent literature on deliberation in transitional justice. Kostovicova shows, counterintuitively, that expressions of ethnic difference play a role in increasing the quality of deliberation across group lines and foster closer bonds of solidarity between people (Kostovicova, 2023: 4). Inter-group interactions and deliberation can have positive impacts and warrant further study, but no one has yet to examine the role of self-interest in these processes, despite scholars of deliberation pointing to its potential importance in good quality deliberation.

We draw on deliberative democratic theory to address this shortcoming. We use Dryzek’s definition of deliberation as ‘communication that induces reflection on preferences, values and interests in a non-coercive fashion’ and deliberative democracy is a way for individuals to come to a binding decision through deliberation (Dryzek, 2002: 76). Ideally, this deliberation should happen under specific circumstances: all individuals should have equal opportunity to influence the process; individuals should give reasons for their arguments; they should treat each other with mutual respect; they should try to find fair terms of cooperation; and, most importantly, no coercive power should be present (Mansbridge et al., 2010: 65–66) Deliberative democratic theory is suitable for the analysis of dialogues across group boundaries, since they are a type of small-group deliberation (Conover and Searing, 2005; Kostovicova, 2017, 2023; Rangelov, 2013; Steiner et al., 2017).

Self-interest in deliberative democratic theory was for a long time viewed much as it is viewed in transitional justice: that the expression of interest had to be focused on the good of all of society. If forced into the ‘language of the common good’, then only the use of ‘we’ was considered beneficial to deliberation, never ‘I’ (Mansbridge, 2006: 125). But scholars found that deliberative processes that aimed to enable understanding, in practice suppressed dissent by discouraging expression of self-interest (Mansbridge, 2006: 119). The result was a deficient deliberative process, where conflicts of interest were suppressed instead of clarified (Karpowitz and Mansbridge, 2005). Studies found that deliberation was more complex and expressions of self-interest have been shown to be beneficial to deliberative processes (Fung, 2003; Mansbridge, 2006).

The transitional justice literature has similarly ignored the role of self-interest in dialogue, because it suffers from a further theoretical and methodological limitation: it does not analyse human interaction as dynamic and sequential. Instead, studies focus on what a person says to another person in a given instant, without looking at what expressions of interest accomplish across a sequence of conversation. In other words, studies capture only moments in conversation, but ignore the ebb and flow of human interaction.

For example, Corry (who sees dialogue across group boundaries as good for processes of conflict resolution, but only if conflict is avoided) quotes a participant– ‘I am not going to tell you who you are. I am going to accept whom you tell me you are’–and argues that this is an individual respecting another and wanting to hear more (Corry, 2012: 72). He goes on to cite similar quotes from other participants, to show that individuals could hold on to their beliefs and respect those of others. This reduces human interaction to one statement made by a single person, out of its conversational context. The (sometimes counterintuitive) effect that the expression of respect can have is lost. Respect is expressed, but there is no examination of what it achieves in conversation. Corry does not show what such a statement does interactionally, what this sequence of interaction looked like before and after this statement and the context that it was made in.

David critiques Corry’s conclusions and instead finds inter-ethnic dialogues to be counterproductive for conflict resolution, but she similarly flattens interaction (David, 2019). She cites numerous examples of individuals’ identities becoming ossified through inter-ethnic interactions, using brief quotes from workshops in Israel/Palestine, such as ‘But in the end You are all Jews. The bottom line is that Jews are Jews, and Palestinians are Palestinians’ or ‘Why don’t You (switch from singular to plural speech) move to Jordan? There is no place for both of us (our people) here!’ (David, 2019: 422) David argues that ‘the rhythmic coordination of gestures transforms participants’ feelings into collective feelings [. . .] where particular essentialized identities are employed to justify and help negotiate one’s own position in the group as a whole’. (David, 2019: 422) Her argument is that this only solidifies group identities and is counterproductive for transitional justice. However, the analysis of the ‘gestures’ that ‘transform’ feelings is done by examining isolated statements made by single individuals, rather than by examining the interaction between individuals across identity lines. There is no exploration of what such statements achieve interactionally or how interactions arrived at such statements.

Studies of post-conflict reconciliation hint at the possibility that self-interest may hold benefits for post-conflict resolution. This is relevant for transitional justice since reconciliation is seen as a goal and normative aspiration of transitional justice (Kostovicova, 2023: 6). Ugarriza and Nussio found that attitudes between ex-combatants and residents of communities hard hit by conflict in Colombia improved most when participants were instructed to ‘express personal experience’, rather than ‘suppress self-interest’ (Ugarriza and Nussio, 2017). However, the authors do not explore why and how this happens. Steiner draws on theories of deliberative democracy and self-interest to further explore the role of sharing one’s experiences in post-conflict reconciliation (Steiner et al., 2017). He finds that sharing of personal stories can aid reconciliation, but only as long as these stories remain focused on the topic at hand (i.e. the conflicts). There is reason to believe that the same holds true for transitional justice, but inter-group interactions have not yet been studied in this manner.

We argue that dialogues are more complex than how they have been studied so far and they require a theoretical and methodological approach that takes into account that human interaction cannot be reduced to a single statement, but it ought to be examined across sequences of interaction that make up a dialogue. To achieve this, we combine democratic deliberation theory and Conversation Analysis methodology (discussed below in more detail) to produce a framework that allows us to track how expressions of interest vary and can shift during an interaction. Drawing on Fung, we expect there to be two stages in this process (Fung, 2003). In the first stage, individuals move from silence to self-expression, thereby asserting statements of interest (Fung, 2003: 344). This can be ethnic interest (as the literature against inter-group dialogues argues); shared interest (as the literature in favour of inter-group dialogues argues); or something else altogether, such as individual self-interest. Expressions of interest in the first stage can take the shape of, for example, complaints, testimonies, story-telling, relating needs, advocacy and the expression of conflict (Fung, 2003; Sanders, 1997). These can be in a single statement by a lone individual or they can be a series of statements by several individuals. Democratic deliberation theory and transitional justice share a commonality in this sense, since transitional justice is often envisioned as a process that provides a space where individuals, even those weak or marginalised, can legitimately speak (Aguirre and Pietropaoli, 2008; Madlingozi, 2010). This first stage is a way of observing the space for legitimate expression in transitional justice.

Within this framework, and reflecting the preoccupation of transitional justice with the peace-promoting and reconciliatory potential of dialogues, we modify the notion of shared interests using democratic deliberation theory. We define shared interests as an orientation towards the common good, defined as a vision of justice that is expressed in relation to a group greater than only an ethnic group, for example, a multi-ethnic state, a region, a generation, or humanity. It is a common good that every member of society has a moral and political responsibility to uphold as a basis for maximising the possibility of peaceful coexistence (Villa-Vicencio, 2006: 393). It is a switch away from ethnic interest.

In the second stage, we can observe the potential shift or shifts in interest. In some interactions, this shift may not happen at all. In others, the type of interest can shift from one type to another, including towards the common good, even after the expression of self-interest. Individuals may constrain the pursuit of their own group interest according to norms of justification; in other words, they can reasonably respect claims of others and they may restrain themselves when others offer compelling reasons for the common good or when they exhibit norms, such as respect, reciprocity and fairness (Fung, 2003: 348). These can be explicit, such as references to a category broader than an ethnic group (all of society, a region, a state, a generation, etc.) or they can be implicit, in the form of clarification, brainstorming, information-pooling, planning and problem-solving (Fung, 2003: 348; Mansbridge, 2006: 108). Allowing for the possibility of interest-shift implies that inter-ethnic dialogues on transitional justice issues are more complex than currently theorised. They may be imbued with potential to foster positive social change, in the form of common good orientations which we can capture if we focus on sequences of talk in inter-ethnic dialogues. This only becomes apparent when inter-ethnic interactions are theorised and analysed as dynamic and sequential, rather than static.

## Methods

We employ Conversation Analysis to follow the sequential nature of conversations. Conversation Analysis is a micro-analytic method for the analysis of turns in conversations. It is premised on the assumption that all speech is orderly and all instances of naturally occurring conversation are equally explainable by general rules (Heritage and Clayman, 2010). Conversations develop turn-by-turn and only by examining these sequences of turns is it possible to better analyse how individuals come to understand each other’s actions and how they construct their responses (Drew, 2015). Conversations are, therefore, regarded as being co-constructed between participants (Drew, 2015). Three key assumptions guide the analysis. First, that talk is action; it is concerned with doing things and taking action (Kristiansen and Grønkjær, 2018). Second, talk is structurally organised, so that single utterances are a part of larger, structurally organised entities. Third, this approach does not assume that content is irrelevant. The nature of the content affects the nature of the interaction and, therefore, warrants examination (Morgan, 2010). For the purposes of this analysis, only some conventions from Conversation Analysis transcription were employed, these are outlined in Table 1. By examining sequences of interaction, we can examine what expressions of interest accomplish in conversation and how they shift. This approach can show that inter-ethnic conversations about transitional justice are more complex than the literature currently suggests. It also addresses the shortcomings of the established literature on transitional justice, which only analyses isolated statements and fails to consider the ebb and flow of human interaction.

**Table 1.** Transcription key (Jefferson, 2004).

[ ] square brackets	Overlapping talk
= equals sign	No discernible interval between turns (one speaker begins speaking immediately as the previous speaker stops)
(0.5) time in parentheses	Length of interval between talk (measured in tenths of a second)
((word)) words enclosed in double brackets	Transcribers comments (for example, to note physical gestures)

The text corpus that is analysed in the study is derived from 12 focus groups conducted in Croatia, Bosnia-Herzegovina, Kosovo and Serbia between 2019 and 2022.^2^ These countries were chosen as typical cases of post-conflict transitional justice where politics and society remain centred around ethnic identities (Hadzic & Tavits, 2020: 1028). The conflicts that followed the breakup of the Socialist Federal Republic of Yugoslavia began in 1991 and continued, with intermittent breaks, until 2001. The four states being studied were most intensely involved in the fighting.^3^ The most visible mechanism of transitional justice in the region was an ad hoc court established by the United Nations that prosecuted war crimes from 1993 to 2017, known as the International Criminal Tribunal for the former Yugoslavia (ICTY). The legacy of the conflict remains pervasive across the region and all of the states are segregated along ethnic lines, to varying degrees (Majstorović and Turjačanin, 2013). The conflict continues to define all of the societies of the region.

Focus groups are well-suited to the study of interactions across ethnic lines because they provide an opportunity for extended discussion and deliberation. They also capture interactional data that allows researchers to investigate how accounts are ‘articulated, censured, opposed and changed through social interaction’ (Cyr, 2017: 1038). Snowball sampling was used for participant recruitment and all groups were composed of individuals of different ethnicities that were involved in the conflict (details on the recruitment process are available in Appendix 1.).

The aim of the snowball sampling was to avoid selecting individuals who work in the field of transitional justice or have extensive knowledge of it. Participants were recruited through three overlapping strategies. The first involved connections established via a gatekeeper known personally by the researcher (Belgrade, Prijedor, Prizren, Sarajevo, Subotica, Zagreb II). These gatekeepers did not participate in the groups. The second approach relied on contacts through NGOs or promotional efforts by local youth organisations (Mitrovica, Prijedor, Vukovar, Zagreb II). The third strategy involved university advertisements, either circulated via email or announced directly by lecturers to humanities and social sciences students (Belgrade, Prishtina, Tuzla, Zagreb I).

Recruitment was challenging due to the high level of segregation in the region, which made it difficult to achieve a balanced sampling approach. While each group included participants from at least two ethnic backgrounds, the proportions varied across groups. This is an inherent issue with snowball sampling; it can lead to a sample skewed towards individuals more willing to openly discuss sensitive topics. In post-conflict research it is frequently used because entire populations often experience some level of marginalisation, making them difficult to access. In addition, snowball sampling can address distrust and fear in conflict environments, as being introduced through a trusted social network increases participants’ willingness to engage with the researcher.

Focus groups are infused with the dynamics of power in wider society and there is a risk that power disparities can impede interaction in the focus group setting (Ayrton, 2019: 323). We, therefore, avoided creating power disparities in our recruitment, for example, by not involving experts on the topic or individuals’ superiors (for example, managers). However, in some groups where local segregation was particularly extreme, the micro-dynamics of power from the local context were also present in the focus groups. Based on the most recent research into focus group methodology, we saw this as an opportunity to enrich our research findings, since it allowed us to examine how power relations affect processes of transitional justice in divided societies (Ayrton, 2019). It provided an opportunity to examine the extent to which deliberative mechanisms can mitigate power asymmetries and allow individuals to deliberate as equals (Lupia and Norton, 2017: 65).

A total of 65 participants took part in the study. Of them, 35 were men and 30 were women; all major ethnic groups were represented; and, the mean age of the sample was 26 (see Appendix 1 for more details on group compositions). The focus on young individuals biases the data since this is a segment of the population that is less interested in politics and transitional justice, even though at the policy level they are seen as crucial to transitional justice processes.^4^ Theoretically, young people are crucial to transitional justice since transformation occurs across generations and it is young people who often catalyse resistance to divisive politics. Better understanding the opinions and needs of the next generation is key to successful processes of transitional justice (Ladisch, 2013). This is particularly acute in the Balkans, where research has shown that youths are socialised into ethnic world views through their education and society more broadly from an early age (Pavasović Trošt, 2018).

The group discussions were moderated by the authors, they were semi-structured and conducted in the local language (Bosnian/Croatian/Serbian), apart from groups in Kosovo that were conducted in Albanian (Prishtina and Prizren) and English (Mitrovica). The Mitrovica group was conducted in English because Serb and Albanian youths (who took part) do not speak each other’s languages and instead communicate in English. The topic of discussion in each group was transitional justice and reconciliation in the region generally, as well as the ICTY and key trials specifically.

## Analysis

The aim of our analysis is to use Conversation Analysis to take into account the dynamic nature of human interaction and examine whether there are shifts in the expression of interest in sequences of interaction. We do so by identifying shifts in interest in sequences of interaction. We observed numerous excerpts where types of interest shifted during exchanges and ethnic interest did not solely result in the exacerbation of ethnic identities or different views of the past. We identified 207 sequences across the 12 transcripts that involved shifts in interest. These sequences ranged in length from brief exchanges (expression of interest in one statement and a shift in the statement that follows) to lengthy discussions over several minutes with numerous shifts. The shifts occur regularly and frequently; on average, 82% of each transcript was composed of sequences that involved these shifts.

Expressions of ethnic interest were observed to result in a common good orientation that only became apparent if the conversation was analysed more completely and sequentially. These expressions of interest and shifts served three distinct purposes for the common good: they helped build a basic understanding of an outgroup; they provided recognition of victimhood; and, they advocated minority interests. Below are typical examples of each, drawn from the corpus of textual data. They were selected because they are representative of others in the category. Sequences analysed in Conversation Analysis often cover numerous pages of transcripts, which makes them difficult to present. The examples were therefore also selected because they are short but still show how interest changes in the sequences of interaction.

### Creating a basic common understanding of an outgroup

In the example below from Mitrovica, Kosovo, it is possible to see how the expression of ethnic interest can be the spark for a common good orientation, as opposed to polarisation as has been previously argued (Corry, 2012; David, 2020; Fisher, 2001; Saunders, 2000). Mitrovica is a strictly segregated city, where Albanians and Serbs have little to no routine contact with each other. It is a focal point for ethnic tensions. This group was, as expected, tense and interactions were often antagonistic. The three male, Albanian participants were often confrontational and accusatory towards the single female, Serb participant. However, as the example below shows, even in such contexts, interactions can turn towards the common good.

What precedes the passage below is the moderator asking the group how inter-ethnic relations can be improved in Kosovo. All three of the Kosovar Albanian participants quickly responded by saying that Serbia had to first accept responsibility for the conflict in Kosovo; an ethnic and antagonistic view of conflict resolution. The Albanian participants then go on to discuss the killing of Adem Jashari, a prominent war time hero for Kosovo Albanians, and his family. Jashari is the most esteemed hero in the eyes of the Albanian Kosovar public; he has come to signify Kosovo’s armed struggle for independence (as opposed to the legacy of peaceful resistance promoted by Ibrahim Rugova during the 1990s); and, he is central to Kosovo Albanian collective memory constructions of martyrdom (Krasniqi, 2014: 153). Jashari, and the Jashari family, exhibit ethnic interest in an antagonistic fashion and the victimhood of one side only:

The key shift in the passage from the first stage, where ethnic interest is expressed, to the second stage, where a common good orientation occurs, starts with an accusation on ethnic grounds that is questioned. Afrim says that Jelena has to accept ‘her fault’, but once she questions that, first Valon and then Afrim are quick to correct themselves and to clarify that they mean her country’s fault. In Conversation Analysis terms, this is a repair and it is significant for transitional justice (Schegloff et al., 1977). Repairs correct ‘interactional errors’ – mistakes in the attempt to speak appropriately to particular recipients under particular circumstances – in order to return an interaction to a neutral footing (Drew and Heritage, 1992: 172). Both Valon and Afrim realised that what they did was problematic, since it broke an interactional norm: that of laying blame on an individual based solely on group belonging (that it was Jelena’s fault because she was a Serb). They are attempting to correct this interactional error and through that process they gained a better understanding of Jelena and her views. This is significant because it is not only a self-repair concerning two individuals engaged in a dialogue, instead it is a repair initiated by a third party, showing a group effect at play. Jelena questions the ethnic group categorisation and forces the others to see her as an individual, as a result of the social interaction of repair.

Following this shift, we see a sequence of clarification and information pooling; ways for people to access a broader range of perspectives and thus become better informed (Fung, 2003: 344). Afrim is now relaying to Jelena why they struggle to talk about other topics; Jelena validates their trauma ( ‘that is not good to hear’); Valon says that the past is difficult for them to deal with; and finally, in the most explicit expression of common good orientation, Jelena says that it is not easy for any of them, meaning young people living in the aftermath of the conflict. The information pooling is significant because it is a sign of high-quality deliberation and it results in fair and legitimate decisions (Fung, 2003: 348). It exhibits reasonableness to participants engaged in deliberation by pooling together of perspectives to reach conclusions (Fung, 2003: 344). Jelena’s validation is significant because it is a way for individuals to show that they understand each other’s positions and are thus attempting to create a shared understanding of the world they inhabit (Jaramillo and Steiner, 2014: 13).

The shift from ethnic interest to a common good orientation occurred through Jelena’s questioning and the group’s subsequent clarification, information pooling and validation. This process allowed the individuals to gain a better, although basic, understanding of each other’s lived experiences and views of the recent past. This process creates what Quinn refers to as ‘thin sympathy’, which enables the development of a more durable transitional justice process (Quinn, 2021).

### Recognition of others’ victimhood

The expression of ethnic interest can spark a conversation that results in a common good orientation when it allows individuals to express their victimhood and for that victimhood to be recognised. Recognition of victimhood is seen as a key component of transitional justice processes and it continues to receive attention in research, policy and practice (Clarke, 2009; Lawther, 2021; McEvoy and McConnachie, 2013). Recognition of victimhood can give individuals voice or agency (Orentlicher, 2018: 19; Robins, 2017; Ross, 2003: 333; Waterhouse, 2009).

The example below is from Zagreb, Croatia. Zagreb is large city where ethnic differences are not as pronounced as in the rest of the region. As some ethnic minority participants noted, in Zagreb it is difficult to tell who is from a minority and who is not, since everyone speaks with the same accent (from Zagreb), everyone supports the same local football teams (sometimes used as a marker of ethnic affiliation) and few youths are religious enough to use that as an ethnic marker (this was discussed in some detail in the other focus group in Zagreb). Often, surnames or family heritage are the only markers of ethnicity. This group was very open in their discussion and most participants either knew each other or were known to each other through shared social circles. They spoke freely and openly from the very beginning. Even when they did not agree, the disagreements were respectful and laced with humour. In the example, the moderator asked participants what they thought about the Karadžić trial and the resulting interaction leads to the recognition of victimhood of a participant whose ethnic group was the target of genocide.^5^

Amar begins his expression of ethnic interest by referring to himself as an ordinary citizen and a member of the ethnicity against which genocide was committed. The first stage expresses ethnic interest, but this ethnic interest is qualified. Amar identifies himself as a member of an ethnic group, as an ordinary citizen (meaning a citizen of Croatia) and specifically not as a lawyer. He is being careful to make it clear that he is expressing ethnic interest and not conflating that with some other categorisation (for example, citizens of Croatia or legal professionals). He thus identifies himself to the others as a victim based on group identity, by saying that he is a member of the group against which genocide was committed.

Shortly thereafter, Emir raises the issue of the legitimacy of the ‘Hague Tribunal’ as a topic (Line 10). Emir is referring to the International Criminal Tribunal for the former Yugoslavia (ICTY). At this juncture, the conversation in the group is not dissimilar to other conversations where the legitimacy of the ICTY was raised. For example, in the Prijedor (Bosnia-Herzegovina) focus group, Serb and Bosniak participants expressed an opinion that the ICTY was seen as biased towards certain ethnicities. Exactly which was left undefined, but one’s own ethnicity was implied. This is reflected in survey data, where respondents across the region feel the ICTY punished one’s own ethnic group too harshly and others not enough (Kolstø, 2011). Conversations about the legitimacy and fairness of the ICTY involve expressions of ethnic interest.

Scholars often interpret expression of ethnic interest in discussions about the ICTY as a failing of the institution. They acknowledge that the Tribunal provided some kind of justice by punishing significant numbers of perpetrators who were key drivers behind the violence, but they argue that the Tribunal allowed groups to retain their own understandings of the conflict (Stover and Weinstein, 2004); that it entrenched and polarised nationalist views of the conflict (Orentlicher, 2018); and that it hindered efforts to diffuse ethnic tensions (Snyder and Vinjamuri, 2003). Our insights contradict these studies, but this only becomes apparent once the full sequence of interaction is analysed.

Emir’s statement about the ICTY receives a reply by Dejan, a Serb, who asked a question for clarification (Line 12). This initiates a discussion over the ICTY and its foundation, including that all states in the region agreed to its foundation. Unlike what the studies cited above would expect to find and unlike the example in Prijedor, where a similar discussion remained focused on ethnicity, here the orientation shifted to the common good when participants discussed the ICTY generally, its foundation and its use to the region. This exchange continues with brainstorming, clarification, questioning across ethnic lines for several minutes, and finally concludes with a condemnation of nationalism in media reporting on the ICTY in Croatia and the region.

This shift is significant because it shows that discussions of the ICTY framed by ethnic interest do not necessarily entrench positions. Ethnic interest is not static and in the example above it does not become cemented. The discussion about the past–that is made possible by the ICTY’s work–can result in a shift towards the common good that is preceded by statements of ethnic interest. This type of discussion and confrontation with the past is unexpected and can be understood as a deviant case, given how recent the conflict was and the highly polarised group narratives in the region (Gordy, 2013). Discussion about the past with diverse audiences outside of the courtroom, as presented above, can be interpreted as a positive effect of war crimes trials (Rangelov, 2013: 48). Such discussions are a way that trials enact social change by encouraging public debate that can challenge and change exclusivist narratives of nationalism (Rangelov, 2013: 46). It is not guaranteed that people discussing the ICTY’s work will arrive at such an outcome, but trials foster conversations that are imbued with such potential. It is also possible that where such discussions take place matters, for example, seeing more of an effect in a small group setting. The potential for such change only becomes apparent when the sequential nature of human interaction is considered appropriately, both theoretically and methodologically.

### Advocating minority interests

Expressions of ethnic interest can also help advocate or promote minority interests that serve a function for the common good. They do so by advocating for ethnic minority rights in a context where minority rights may be threatened. They aid formulation or discovery of the common good within a multi-ethnic state. The shift does not only have to move from ethnic interest to a common good orientation. It is possible for multiple shifts to happen in different directions. Below is an example from Prizren, Kosovo, of how a common good orientation can be followed by a shift to ethnic interest and then back to the common good once again. Participants in this group did not now know each other well and it was a very mixed group, but it did not include any Serb participants. It is important to note that there are many different minorities in Kosovo, many of whom do not feel like fully fledged citizens of the state. The interactions in this group were often stunted, but rarely antagonistic. Nevertheless, there were tensions between the Albanians (the majority ethnic group) and other minorities. This highlights the plight of some minority groups, that do not feel seen in society.

The passage begins with an explicit statement of interest oriented to the common good, concerning the problem of nationalism in both Kosovo and the region. However, what Arben, Ilir and Amir go on to construct is a complaint on ethnic grounds. They claim that the interests of one ethnicity, the majority Albanian one, take precedence over their ethnicities. Ilir provides the most striking example of how when Kosovo scores a goal in football, all Albanians of Kosovo are congratulated. This places the focus on ethnic or national identity, rather than civic identity. The nominally multi-ethnic state is thus portrayed to be mono-ethnic.

The participants then continue making a complaint based on their own various ethnic interests, but a closer reading reveals that the expressions of ethnic interest are oriented towards the common good. This becomes apparent towards the end of the exchange, when Arben states that ‘we all are building this thing together’ and he asks why there is a need to always be something else (meaning an ethnicity), other than a Kosovar (referring to all citizens of Kosovo, as an overarching national category). Such use of ethnic interest advocates for the rights of the non-majority in a context where those rights may be threatened (Landau, 2017). The expression of ethnic interest in this context promotes liberal democratic norms of civic nationalism and is therefore an example of a common good orientation.

These individuals are expressing ethnic interest to discuss the common good of their multi-ethnic state and of being a Kosovar. These expressions foster better deliberative processes in a manner that is directly relevant to transitional justice. By advocating for their minority rights, participants are seeking equal political status in a society with a deep cross-cutting cleavage (Gutmann and Thompson, 2000: 27). This interaction is a dispute over the definition of community. Arriving at a shared definition of the community they inhabit can result in higher quality deliberation (Mendelberg and Oleske, 2000: 187). The expressions of ethnic interest by individuals are a way for non-majorities to express the collective interests of the group and to better form conceptions of the group, of the community more widely and of justice (Mansbridge and Benhabib, 1996: 58). Transitional justice policy suggestions aim to do exactly this: promote understanding between groups by strengthening the claims of the marginalised by offering opportunities for open-ended discussions, including the status of minorities (Chapman, 2009). This is a complex and messy process that requires compromises and reflection on new forms of citizenship and inclusivity (Chapman, 2009). Our analysis captures this process empirically because it theoretically and methodologically accounts for its complexity.

### Failed shifts

Shifts in interest were common in the transcripts. However, at times expressions of ethnic interest did not result in any kind of shift. Such interactions were stunted and brief. An example from Prijedor (Bosnia-Herzegovina) is a typical case of this. Prijedor is a highly segregated city (composed of predominantly Serbs and Bosniaks) in the Republika Srpska part of Bosnia-Herzegovina. During the conflict the ethnic cleansing in the area was so extensive that in the eyes of the ICTY it met some of the requirements for genocide.^6^ This particular focus group was formed of three male Serb participants and one female Bosniak participant. There was no direct or open confrontation in the group, at least not between participants, but the conversation was muted. Whenever a sensitive topic was touched upon, the group would fall silent.

Ethnic interest is implied throughout this exchange. First Nikola mentions in line 7 that some were held to be more responsible than others by the transitional justice process and Dragan agrees line 9. This is a common complaint that transitional justice targeted one ethnicity more than another. Adisa follows, by saying that not only a single side ought to be judged. Again, she is constructing a complaint that one side was singled out. The implication is that this is not the same side that Nikola and Dragan were referring to. These expressions of ethnic interest were common in the group and were made explicit later when Dragan directly asked the moderator why they were only focusing on the Serbs, since the Bosniaks also committed crimes, but in his eyes were not punished for them. The effect is that the sequence of interaction ends and there is thus no further shift. There is simply no further conversation and no opportunity for the interest to shift towards the common good.

## Alternative explanations

Power relationships and norms in segregated post-conflict communities present three plausible alternative explanations that account for shifts in interest. First, it is possible that individuals are shifting their expressions of interest due to power relations in the focus groups. Focus groups involve inherent power imbalances between participants, as well as the researcher (Smithson, 2000). Pre-existing group norms outside the focus group can privilege certain individuals with higher status (Kitzinger, 1995). This allows the advantaged and privileged to encourage or ensure the compliance and acquiescence of others (Lukes, 2021: 284). In a segregated post-conflict context, this could mean that a member of a group lacking privilege (for example, a victimised minority) may choose to conform to views of the other side and thus be observed to have an interest in a common good present by the privileged participant.

This explanation is not convincing in comparison to our own due to three reasons. First, we took care to reduce power imbalances in groups. For example, participants were from a similar age group, there were no experts on the topic in groups and we avoided choosing individuals with any kind of professional acquaintance that could impact their responses. Second, if power imbalances were present, then the focus group literature suggests we would observe silences, rather than the responses we observed (Wood and Ristow, 2022). For example, we expected this might occur in Prijedor and Mitrovica, both highly segregated communities where a female participant from one ethnicity took part with several males from the other ethnicity. Silences were observed, but so were the types of dynamic interactions that result in interest shifts, as outlined above. Third, these interactions did not seem inhibited by power imbalances, since they involved elements of well-functioning communication, interpreted in different ways. Using Conversation Analysis we observed repairs used to correct interactional errors so that interaction was kept on a neutral footing (Drew and Heritage, 1992: 172); information pooling and brainstorming were evidence of high-quality deliberation (Fung, 2003: 348); and, we empirically observed the development of ‘thin sympathy’ (Quinn, 2021). All of these indicate that individuals were not merely conforming to others’ views due to power imbalances.

The second possible explanation is that we are observing deliberative restraint. This can be a way for individuals to maintain harmonious relations through the use of ambiguity and ritual politeness in interactions (Mac Ginty, 2014; Ware and Ware, 2022). It is also a reflection of empathy that can result in everyday peace (Mac Ginty, 2021). The strategy can be beneficial to individuals in precarious situations, since it can help them survive, maintain dignity and rebuild a sense of normality (Eastmond, 2010: 12). Deliberative restraint can be observed by individuals actively disengaging from interactions in order to maintain peace (Ring, 2006: 163). This was at times observed, for example in the Prijedor group, and the many ‘don’t know’ statements in the transcripts could also be interpreted as deliberative restraint (Sokolić, 2023). But all groups also saw frequent and unambiguous expressions of interest. This is far from restraint, it is active assertion of claims.

Third, it is possible that, given the focus on young people in the study, power relations play a minimal role in interactions, but instead norms of moral authority and empathy hold more sway. Young people may feel they are not entitled to comment on political affairs, such as transitional justice (Bourdieu, 1984: 399). Especially in the context of societies where war veterans and victims have discursive hegemony over conflict related topics, it is possible that young people feel that transitional justice is restricted to a closed group of individuals, which does not include them (Laurison, 2015: 944). Once an individual makes an expression of self-interest, others may not feel entitled to challenge it. Again, as in the above explanation, there were numerous instances where claims were challenged and where self-interest resulted in a common good orientation based on questioning (Mitrovica perhaps being the starkest example). These explanations hold explanatory potential, but it is not as strong as the framework we propose through the combination of deliberative democratic theory and Conversation Analysis methodology.

## Conclusion

Transitional justice practice and scholarship view expressions of self-interest, especially in the form of group interest, as inherently counterproductive to the process of transitional justice (Demirel, 2023). They argue such expressions entrench divisive ‘us’ versus ‘them’ relations (David, 2020). Studies come to this conclusion because they analyse expressions of self-interest in isolation, without looking at what such expressions accomplish across a sequence of interaction, such as a conversation or discussion. We argue that human interaction and social change associated with transitional justice is more dynamic and that expressions of self-interest are more complex than previously theorised. Expressions can shift in interactions between individuals across group identity lines and in doing so they can result in orientations towards the common good that reach beyond group identities. This type of orientation helps foster transitional justice because it engenders a universal vision of justice. It shows that individuals are prepared to discuss a vision of justice that goes beyond the ‘selfish’ interests of a group.

We theoretically capture these shifts by drawing on works that have re-conceptualised the role of self-interest in processes of democratic deliberation (Fung, 2003; Mansbridge, 2006; Mansbridge et al., 2010). These show that self-interest can result in higher quality deliberation since it does not suppress dissent and aids in interest clarification. We apply Conversation Analysis methodology to capture these shifts, because a sequential analysis of expressions of interest enables us to examine interaction rather than only isolated statements of interest. We apply this novel framework to focus group data and find that expressions of interest shift and can be observed to serve three distinct purposes for transitional justice. First, they help build a basic understanding of an outgroup; second, they provide recognition of victimhood; and third, they advocate minority interests.

It is difficult to tell what the lasting effects of these interactions are. How long do they last? Is there a spill-over effect to others? These are also questions that the deliberative democracy scholarship struggles to answer. There is evidence that these types of small-group deliberative interactions can have a lasting impact on interest in politics (Brown, 2006), political engagement (Smith and Wales, 2000), issue knowledge (Luskin et al., 2002) and policy attitudes (Goodin, 2012). However, most of these results lack clear replications and quantitative studies often lack control groups over time (Van Der Does and Jacquet, 2023: 227). Qualitative studies have, in contrast, shown much more positive and long-lasting changes in opinion, knowledge and general awareness of how politics functions (Van Der Does and Jacquet, 2023: 229). It is also possible that other types of expressions of self-interest might be relevant in other contexts that have yet to be studied. Negotiations of any type can be more productive if they involve expressions of self-interest; from diplomatic negotiations to business meetings. The underlying dynamic ought to remain the same because statements of self-interest can help clarify the needs parties in the process.

Addressing the deep divisions caused by conflict requires long-term projects and brief deliberations most likely have a limited impact on their own. Ugarriza and Nussio, in their investigation of deliberation following conflict, find the effects to be limited and caution that deliberation alone is not enough to overcome structural limitations (Ugarriza and Nussio, 2016). Steiner and his colleagues come to similar conclusions and propose that, if deliberation is to have a lasting effect, it has to be a part of a broader, long-term process that allows for a snowball effect through generations and across society (Steiner et al., 2017: 260). A better understanding of self-interest in deliberation about transitional justice can help form part of this long-term process.

The novel insight that expressions of self-interest are not stable and can result in an orientation towards the common good has two important implications for study and practice. First, these findings advance the literature on intergroup dialogues, especially on transitional justice topics. This scholarship oversimplifies human interaction and consequently ignores the potential for social change to occur through inter-ethnic dialogues about a difficult past. The current scholarship is critical of inter-ethnic dialogues because it is based on a faulty assumption over how human interaction functions and can be analysed. Our approach better takes into account the sequential nature of human interaction that shows that expressions of self-interest are not only dynamic and complex, but that they can be beneficial to processes of transitional justice. Studies that concluded that such statements were detrimental to the process also called for the suppression of self-interest, which is reflected in current practice (Corry, 2012; David, 2020; Fisher, 2001; Saunders, 2000).

The second implication of our insights is on practice. Our findings show that discussions of transitional justice topics across group identity lines should not be suppressed and that they do not necessarily result in the entrenchment of group divisions. Instead, they can result in the positive outcomes we outline above. This is possible when individuals feel they can express themselves freely, including when they feel they can express self-interest. Our findings also show that moderators working in such interactive settings need to be aware that statements of interest can shift and result in a common good orientation, but that this may only happen across a sequence of interaction. This knowledge can help to better steer interactions so as not to suppress or instantly challenge expressions of self-interest. Overall, the findings point to the need to better understand the micro, deliberative dimensions of transitional justice processes that remain poorly understood due to current theoretical and methodological limitations.

## Appendix 1

**Table:**

**Total participants**	65			
**Gender (total number)**	Male 25; Female 35			
**Age range**	18-42^7^			
**Mean age**	26			
	*Bosnia-Herzegovina*			
	Prijedor (4)	Sarajevo (4)	Tuzla (7)	
		*Croatia*		
	Vukovar (5)	Zagreb I (6)	Zagreb II (5)	
**Locations of focus groups (size of group)**				
		*Kosovo*		
	Mitrovica (4)	Prishtina (6)	Prizren (6)	
		*Serbia*		
	Belgrade (8)	Subotica (5)	Novi Sad (5)	
**Total number of ethnicities**	10			
	Bosniak (15)	Serb (14)	Kosovo Albanian (11)	Croat (8)
**Ethnicities represented (number of participants)**	Macedonian Albanian (1)	Roma (3)	Gorani (1)	Bunjevac (1)
	Montenegrin (1)	Turkish (1)	Half Croat/Half Serb (3)	Half Bosniak/Half Gorani (1)

## Notes

1. The fighting occurred in great part (although not exclusively) along ethnic lines. It can be summarised as: in Croatia, Croats against Serbs and Montenegrins (1991–1995); in Bosnia and Herzegovina, between Croats, Bosniaks (Bosnian Muslims) and Serbs and Montenegrins (1992–1995); and, in Kosovo, Albanians against Serbs and Montenegrins (1998–2001). For a more detailed overview, see Baker (2015).

2. Three per country.

3. For an overview, see Baker (2015).

4. For example, see the International Centre for Transitional Justice (https://www.ictj.org/our-work/transitional-justice-issues/children-and-youth)

5. The trial of Radovan Karadžić is one of the best-known cases at the ICTY. Karadžić was the President of Republika Srpska, in Bosnia-Herzegovina, during the conflict. He was a fugitive from the ICTY from 1996 until 2008, when he was captured in Belgrade, Serbia. In 2016, he was found guilty of war crimes and crimes against humanity, as well as genocide in Srebrenica. The crimes targeted Bosniaks and Croats in Bosnia-Herzegovina. In 2019, the Tribunal rejected his appeal against the conviction and sentenced him to life in imprisonment. Second only to Slobodan Milošević, Karadžić was the most powerful political figure on trial at the ICTY.

6. See http://www.icty.org/x/cases/karadzic/tjug/en/160324_judgement_summary.pdf

7. Aside from the participant that was 42 years old, all other participants were 35 or younger.
